# Nutritional Characteristics Assessment of Sunflower Seeds, Oil and Cake. Perspective of Using Sunflower Oilcakes as a Functional Ingredient

**DOI:** 10.3390/plants10112487

**Published:** 2021-11-17

**Authors:** Ancuţa Petraru, Florin Ursachi, Sonia Amariei

**Affiliations:** Faculty of Food Engineering, Stefan cel Mare University of Suceava, 720229 Suceava, Romania; ancuta.petraru@fia.usv.ro (A.P.); florin.ursachi@fia.usv.ro (F.U.)

**Keywords:** sustainability, sunflower seeds, sunflower oil, sunflower oilcakes, nutritive parameters, classification, amino acids profile, fatty acids composition

## Abstract

Ample amounts of by-products are generated from the oil industry. Among them, sunflower oilcakes have the potential to be used for human consumption, thus achieving the concept of sustainability and circular economy. The study assessed the nutritional composition of sunflower seeds, cold-pressed oil and the remaining press-cakes with the aim of its valorization as a food ingredient. Sunflower oil contains principally oleic (19.81%) and linoleic (64.35%) acids, which cannot be synthetized by humans and need to be assimilated through a diet. Sunflower seeds are very nutritive (33.85% proteins and 65.42% lipids and 18 mineral elements). Due to the rich content of lipids, they are principally used as a source of vegetable oil. Compared to seeds, sunflower oilcakes are richer in fibers (31.88% and 12.64% for samples in form of pellets and cake, respectively) and proteins (20.15% and 21.60%), with a balanced amino acids profile. The remaining oil (15.77% and 14.16%) is abundant in unsaturated fatty acids (95.59% and 92.12%). The comparison between the three products showed the presence of valuable components that makes them suitable for healthy diets with an adequate intake of nutrients and other bioactive compounds with benefic effects.

## 1. Introduction

Large amounts of biodegradable waste and residues are produced and discarded every year from the food industry. These residues have high biochemical and chemical oxygen demand. For this reason, untreated waste harms the microflora [1]. Food waste with a high fat content is susceptive to oxidation and thus harmful for microflora due to continuous enzymatic activity, which accelerates spoilage and limits technological possibilities of disposal. Environmental protection must be the first priority of international politics [2]. Nowadays, due to the rapid expansion of the human population and current environmental issues (over-exploitation of resources, degradation of environment), it is necessary for a transition to the circular economy and the development of new strategies to minimize food waste during the supply chain [3,4]. Moreover, problems related to cost disposal and the use of by-products have become an increasing challenge [5]. Conventional methods for waste disposal and valorization are incineration, aerobic fermentation, composting, fertilizer and feedstuff [6]. Alternative solutions consist in extracting the maximum value from waste, hence the bioactive compound and re-circulating them in the process, making value-added products and thus creating the concept of “waste = food” [7,8].

Oilseeds are mostly used as a source of vegetable oils. After the extraction process, large amounts of residues and by-products are available. The use of these permits the achievement of effective waste utilization and the successful realization of the circular economy concept [9]. Possible valorization methods of the oilcakes involve their use in animal diets as feeds, compost, a substrate in the production of enzymes, antibiotics and biosurfactants and in the recovery of bioactive compounds for further use in the production of new value-added products [6,10].

Sunflower is a plant from the *Asteraceae* family, *Helianthus* genus and more than seventy species are known worldwide. The origin of the name derived from the aspect of the plant which resembled a sun and the fact that it rotates after the sun’s rays [11,12]. Globally, sunflower seeds are ranked as one of the most produced oilseeds crops alongside rapeseed, soybean and cottonseed [13]. Their composition and nutritive values depend on numerous factors, namely genotype, soil type, agricultural practices, climatic and processing conditions [14]. Two types of sunflower seeds are known, namely the oil-producing seed and the ones used for confectionary purposes. The first is black with a thin hull (lignin and cellulolytic materials) that adheres to the kernel and represents 20% of the total weight. Originally, the seeds contained 25% oil but by modern plant breeding methods [15,16] (induced mutation, hybridization, molecular breeding) new sunflower hybrids, in which the oil content was increased to 40% [11,17,18], were created. The seeds are a source of dietary fibers, unsaturated fatty acids (more linoleic than oleic), antioxidants, flavonoids (quercetin, luteolin, apigenin and kaempferol) amino acids, proteins (up to 20%), vitamins (E, B, folate and niacin) and minerals (principally calcium, copper, iron, magnesium, manganese, selenium, phosphorous, potassium, sodium and zinc) [19,20]. The amino acids profile includes glutamic, aspartic acids, arginine, phenylalanine, tyrosine, leucine, methionine and cysteine. The content in fatty acids varies up to 31%, being higher than the other oilseed such as safflower, peanut, soybean, sesame and flaxseed [21].

The oilseeds are mostly used as a source of vegetable oil with unique physicochemical properties [22]. The traditional extraction techniques involve the use of a mechanic press (hot or cold pressing) or solvents [23,24]. Sunflower oil, due to its easy accessibility and numerous health benefits (maintenance of low cholesterol and low-density lipoprotein levels in the human body, antioxidant, anticancer, antihypertensive, anti-inflammatory, skin protective and analgesic), is widely preferred in Europe, Mexico and several countries of South America. After extraction, it remains liquid at room temperature and has a shelf-life of over one year at 10 °C and in darkness [11,25]. The major components are linoleic (59–65%) and oleic acids (30–70%). These represent 48–78% of the total fatty acids profile. There is also a small percentage of palmitic and stearic acids (15% for both fatty acids) present [26,27]. Sunflower oil is also rich in vitamins (important role in the good functioning of the skin, nerves and digestive system), minerals (role in the enzymatic and metabolic processes) and excellent phytochemical such as carotenoids, tocopherols, phenols and tocotrienols with antioxidant activity. The variation of the composition depends on the plant’s species and the extraction methods employed [28,29].

Oilcakes are the principal by-products obtained after the extraction of oil from the seeds. Then they are air-dried to remove the water before storage. Sometimes they are molded into two forms, namely flour (ground material) and pellet. The term is synonymous with press-cake, meal and oil meal. In terms of appearance, sunflower meal has the taste and the smell characteristic of the initial raw material without musty, mold, rancid and foreign smells. The color changes from black to gray [30].

Generally, the meal is used in animal diet as feeds because it is an excellent source of protein and thus produces an increase in biomass [31,32,33]. It can be also used for human consumption. The essential amino acids present in sunflower press-cakes are cysteine, methionine, leucine, valine, isoleucine, tryptophan, alanine and phenylalanine [25]. Regarding the minerals and vitamins, phosphorus, thiamine, nicotinic, pantothenic acids and riboflavin are predominant [26]. The dehulled process reduces the fiber content and increases the protein content [34]. The difference in chemical composition varies depending on variety, growing condition, dehulling and extraction method [35].

The physical characteristics are extremely important in the seeds production chain for designing various agricultural machines and equipment for operations such as planting, harvesting, cleaning, quality assessment, classification, dehulling, milling, packaging, storing and oil extraction [36]. Physical characteristics can be grouped into four categories, namely dimensional, gravimetric, compressive and frictional (angle of response and static friction) [37,38]. Length, width, thickness, area, volume, sphericity, equivalent diameter and projected area are dimensional proprieties and offer information about the shape. Instead, mass, bulk density, true density and porosity are gravimetric properties [39]. The size, shape and density influence aerodynamic proprieties, which are crucial in designing a harvester [40]. Moreover, hulling efficiency is affected by the hull structure, seed size and density. Sphericity, static friction and angle of response play a key role in designing storage facilities, while porosity in designing extraction machinery [41]. When evaluating quality, consumers choose their preference based on texture, flavor and appearance. In order to reduce damaged and defective seeds, a classifier that complies with the quality indicators found should be realized [42,43].

The aim of this study was to investigate the physicochemical properties of seeds, oil and oilcakes and the transformations that take place in the processing stages of the raw material. This study is important to evaluate the losses in the processing and to discover the bioactive compounds that can be extracted from the by-products after oil extraction. This study was also conducted to determine the physical attributes of seeds and kernels in order to classify them into quality classes.

## 2. Results and Discussions

### 2.1. Chemical Composition of the Seeds, Kernels and Hulls

Sunflower seeds consist of kernels and seed coats or hulls. Sunflower hulls represent 21–30% of the seed weight and generally are considered a waste by-product [44]. From the hulls can be recovered valuable phenolic compound and cellulose fibers for the production of green, renewable, biodegradable and edible food packaging material, thus reducing the global plastic production [45]. Another alternative to dispose of them is by transforming the biomass (by pyrolysis, gasification and fermentation) obtaining bio-oils rich in furfural content (a valuable bio-renewable chemical that can be used in the production of biofuels and biochemicals) [46] In our study, the proportion of hulls calculated for the sunflower seeds fell into the range from 13.67% up to 43.47%. Values depend on variety, environmental conditions, seed size and oil content [47]. The lower values can be the result of the continuous effort to increase the seed’s oil content [40]. The hulls contain a low percentage of proteins (7.82%), lipids (8.81%) and ash (2.45%) according to Table 1. Similar values were found by other authors [40,46,48], values ranged within 3.48–12.40% for moisture, 2.3–9.45% for oil, 5.36–7.36% for protein and 2.1–4.11% for ash.

Nutritional characteristics of mature and sun-dried sunflower seeds, hulls and kernels are summarized in Table 1. The findings showed that seeds contain on average 33.85% proteins, 65.42% lipids and 2.73% ash, most of which are found in the kernels.

In comparison with other species, the seeds are rich in crude fat, due probably to the breeding techniques (induced mutation, hybridization, molecular breeding) used to increase the oil content. Various studies about the chemical composition of high-oleic sunflower seeds showed that the fat content varies in the range of 37.47−54.06% [49,50,51].

The ash content found in various high-oleic seeds ranged between 2.68–4.87%. The findings were lower than those reported, differences being attributed to genetic factors and geographical conditions [52,53].

Compared to the other nutrients moisture content is the most important factor for the prevention of insect infestation and diseases. Moreover, moisture content affects the physical properties of sunflower seeds. With the increase, the spatial and gravimetric also increased [39,54,55]. Moisture values found in the literature ranged between 2.5−6.32% and 3–3.2% in whole and dehulled seeds, respectively [35,56]. The higher values were found in hulls because they have a higher water absorption. Further, low moisture in kernels can be explained by the content in oil because the two liquids are immiscible [40].

When the dehulling process is applied in kernels the protein content increases up to 23.73% and ash content up to 3.31%. The findings are in accordance with those reported by other authors [35,57]. While opposite results were observed for lipid and moisture parameters. The findings indicate that the dehulling process can contribute to improving quality and reducing undesirable characteristics. However, hulls facilitate the oil extraction process. In this case, an amount of the latter should be left, but in small proportion so as not to compromise the oil quality [35].

The result obtained for kernels were similar to those obtained by other authors [58,59,60].

### 2.2. Classification of the Sunflower Seeds

Lipids are predominant in the kernel’s cellular structure and for this reason, their mass can be considered a potential quality parameter [40]. To investigate the latter, the coefficient of correlation between the size (L, l, W, w, T, t-length, width and thickness of the whole seeds and kernels respectively), shape (ψ, ψ_k_-sphericity of the whole seeds and kernels respectively; D_e_, D_ek_- equivalent diameter of the seeds and kernels) and gravimetric parameters (M, m- mass for the seeds and kernels) was calculated for a sample of 145 unsorted seeds. The results are given in Table 2. All the correlations were significant at *p* < 0.05. The L/W, L/T, W/T ratios indicate that length is more related to width and thickness, however, width is strongly related to thickness. Kernel’s length, width and thickness ratio showed a low correlation with each other. The correlation coefficient for L/l, W/w and T/t ratios indicates the fact that bigger seeds when dehulled give bigger kernels. To investigate the potential correlation with the mass, there were calculated all the ratio combinations with the spatial parameters of the seeds and kernels. Mass is more related to thickness and width in the seeds and with length in the kernels. A moderate relationship between the spatial characteristics of the seeds and kernel with the mass of the kernels was found. Moreover, strong relationships were found between the seeds and the kernel’s mass (r = 0.97) and between the equivalent diameter and the kernels’ and seeds’ mass (r = 0.84). However, the equivalent diameter is hard to obtain because it depends on the length, thickness and width. Overall, it can be concluded that there is a high linear relationship between the hulls and seeds’ mass.

Considering the values obtained, the mass can be used as a parameter for the classification of sunflower seeds in three classes. The mass boundaries for each class were taken from Munder, 2017 [40]: for class I, m ≤ 0.045 g, for class II between 0.045–0.070 g and m > 0.070 g for class three. A proportion of 13.08% of the total sunflower seeds sample enters in the first class, a percent of 55.23% in the second and one of 21.68% in the third. Based on the lack of significance found for M/m Gupta and Das [55] choose to classify sunflower seeds based on their length. On the other hand, Santalla et al. [61] despite finding M/m the highest significant combination, choose a classification based on the length and did not justify their decision.

### 2.3. Size, Shape and Gravimetric Properties of Sunflower Seeds and Kernels

The dimensional, geometric and gravimetric properties of the four seed and kernel categories are presented in Table 3. ANOVA analysis indicates that with the increase in mass, all the shapes and spatial dimensions of the sunflower seeds and kernels have expanded significantly (*p* < 0.05%) giving longer, wider, thicker, rounder and heavier seeds and kernels. The significant increase in bulk density and decrease in porosity with classes is correlated with the sphericity because rounder objects tend to occupy more equally the space within a given volume. As the thickness was lower than the width in both kernels and seeds, they can be described as having compressed oval bodies [40]. Further, the dimensional properties are important for determining the seed processing machines’ aperture [62].

The bulk density (p_b_) through the three classes of seeds varied from 395.23 Kg/m^3^ to 425.47 Kg/m^3^, while kernel’s density (p_bk_) varied from 414.81 Kg/m^3^ to 598.08 Kg/m^3^ . Kernel’s values were higher than those of seeds, due probably to the hulls which are bulkier and provoked lower values for the mass per unit volume occupied by the seeds [55]. This characteristic depends on the distribution of seeds after shaking and the shape of the single particles, The more compacted and shaken the seeds are, the higher the values that are found for bulk density [63].

True density values for kernels (p_tk_), namely 1068.60 Kg/m^3^ to 1079.69 Kg/m^3^, were higher than those found for seeds (p_t_), namely 708.07 Kg/m^3^ to 650.33 Kg/m^3^. The finding indicated that seeds will float in water while kernels will sink [39]. Furthermore, according to this information, the separation of the hulls can be carried out by blowing air instead of floating in water [64]. The decrease in overall true density with classes may be due to the decrease in water absorption caused by the oil molecules and the increase in proteins [65].

Porosity decreased through classes from 61.58% to 44.03% and from 43.18% to 34.58% for kernels and seeds respectively. The porosity is important during the drying process because indicates the resistance of the seeds to airflow [62]. Kernels’ sphericity (0.50) was lower than those of the seeds (0.53), making the seeds closer to the shape of a sphere than the kernels. However, the ψ found was relatively low indicating the difficulty of the seeds to rotate easily during handling [66]. Moreover, sphericity near the value of 1 shows a higher tendency to rotate about any of the three major axes. This information is important in designing seed hoppers [62].

Values found by others authors for the seed’s bulk density, true density and porosity ranged between 267.03–710 Kg/m^3^, 444.39–902 Kg/m^3^ and 31.3–54.93% respectively. While those for kernels ranged between 535–582.50 Kg/m^3^, 1015–1250 Kg/m^3^ and 45.4−51.19% [37,39,40,41,54,55,61,67,68].

All seeds categories are longer, wider and thinner compared with the Modern sunflower variety [55]. In comparison with the sunflower hybrid F1 from cultivar PR65H22, the seeds presented similar lengths, but they were thicker and wider [40]. Moreover, the findings showed smaller, wider and thicker seeds than Trisum 568 sunflower genotype [61]. Compared with the two sunflower hybrids (ACA 884 and Paraiso 20) selected by de Figueiredo [41], the unsorted seeds were longer, wider and thicker. The opposite was observed when unsorted seeds were compared with the PSH-996, Shamshiri and the P64H41 varieties [37,39,54]. From the six varieties studied by Cetin,2020, [49] Transol and Colombi showed higher dimensional values, while Tunca presented a similar dimension, except for the length, which was longer.

For the unsorted category, the equivalent diameter, sphericity, volume and surface area values were higher than those reported for the PSH-996 variety. However, they were lower than those reported for Shamshiri, P64H41, LG5582, Transol, 63MM54, P64LC53, Colombi varieties. The three categories of sunflower seeds presented slightly higher sphericity, diameter and volume values than those reported for the PR65H22 variety.

### 2.4. Sunflower Oilcakes Characterization

The physical, chemical and functional properties of the two types of cold-pressed sunflower oilcakes are reported in Table 4. The analyzed sunflower oilcakes have different shapes, namely pellets (SFOC/PE) and cake (SFOC/C). The nutritive composition of the sunflower oilcakes can differ considerably depending on the quality of seeds, extraction technique and storage parameters. All the findings fell in the range reported by other authors [69,70,71]. No significant difference (*p* < 0.05) in the moisture and protein content was found between the two samples. SFOC/C presented significantly higher ash and crude fiber values, but lower fat content than SFOC/PE.

The moisture content is an important factor to maintain oilcake stability for long periods of time [72]. A level below 12% is considered safe for storage because it prevents the rapid growth of mold [62]. The values obtained were 8.75% for the meal pellets and 8.93% for the meal cake. The values were relatively similar to those reported for soybean, rapeseed, sesame and flaxseed. Much lower values were found for hemp seed and pumpkin [69,72,73,74].

Oilcakes should be admitted for human consumption when there is an equilibrated proteins and lipids ratio, optimal values for the human body should be 20–25% and 3–5%, respectively [75]. In our case, the fat amounts are too high so direct consumption is impossible. Thus, sunflower oilcakes are destined for the extraction of bioactive compounds.

The total dietary fiber content in the two sunflower press-cakes was high (31.88% for SFOC/PE and 12.64% FOR SFOC/C). The findings met consumers’ demands for fiber-rich food. In addition, fibers have numerous beneficial effects (increase laxation and decrease blood pressure, cholesterol level, reabsorption of bile acids and starch digestion) [76,77,78,79].

Water retention capacity (WRC) offers information about the degradation of the molecular components by measuring the amounts of solid components released from proteins and other molecules. The WRC for the two sunflower press-cakes was 4.67 g/g for the one in pellet shape and 5.51 g/g for the cake, the difference between the two was not significant (*p* < 0.05) and was probably due to the moisture and protein contents. The same results were found by other authors and ranged from 2.10 g/g to 4.48 g/g [80,81].

The capacity to absorb oil and water is determined by the non-polar and polar amino acids composition, respectively. The oil holding capacity of SFOC/PE was slightly higher than those of SFOC/C, but the difference was not significantly different (confidence level of 95%). A small amount of lipids and moisture increases protein solubility and thus the absorption capacity of the oilcakes [80]. In other studies, values for OHC that ranged from 0.71 g/g to 2.2 g/g [80,81,82,83,84,85] were found.

Water holding capacity (WHC) is measured by the amount of water absorbed by the molecules. The parameter is important for determining the storage conditions. The difference found between the two oilcakes was significant (*p* < 0.05) [79]. SFOC/C presented higher moisture content that provoked a reduction of the degradation of the molecule and hence the reduction of the parameter. Another possible explanation for the highest values found in SFOC/PE refers to the high content of dietary fibers [85]. WHC values found in our study were similar to those reported by other authors, which ranged from 0.71 g/g to 3.27 g/g [80,81,82,83,84,85].

The bulk density (BD) of the two oilcakes was not significantly different from each other. The index decreased with moisture and increased when lipids content decreased. BD is an important property in the packaging and handling processes in the food industry. It is a measure of flour heaviness and depends on the attractive intermolecular forces, particle size and number of positions in connection [72,86]. Other values found in literature ranged from 0.592 g/mL to 0.741 g/mL [80,87]

Emulsion capacity (EC) is the property of mixing two immiscible liquids (water and oil). Emulsion stability (ES) measures the amount of water released from the emulsion over time. The parameters are closely related to protein surface hydrophobicity that allows a better molecular anchorage of the oil–water interface and thus more stable emulsions [72]. SFOC/C presented a lower value for EC and ES that might be due to its lower amount of hydrophobic amino acid. In the literature, higher values for EC (49.09–53.2%) and ES (48.23–50.45%) [88,89] were found.

In terms of color, the SFOC/C was significantly lighter (L* = 46.29) than SFOC/PE, with higher redness (a* = 1.57) and yellowness (b* = 8.90) values. Compared to the sunflower oil analyzed by Grasso et al. [85] our meals are lighter, less red and more yellow. The color of the products obtained in the previous study was influenced when the content of 18% sunflower oilcake was added, the color becomes browner.

The values of all the nutritional parameters analyzed for the whole seeds and press cakes presented differences with each other (*p* = 95%). Sunflower seeds had a higher caloric value than the oilcakes due to their higher lipid content. In the meals, the proportion in seeds reached 65.42% and decreased to 14.16–15.77%, once oil extraction by cold pressing was realized. Cold extraction is the most preferable method to obtain high-quality virgin oil. However, an important oil fraction remains in the press-cakes and increases their nutritional values because it provides the whole health benefits to these by-products [90]. The remaining parameters (ash, protein, carbohydrates) increased in the oilcakes as a result of oil removal [90].

Fat content in press-cakes was still high (15.77% in pellets and 14.16% in cake) and needed a further re-extraction. This can be realized with solvents or hot temperature pressing. The first allows to obtain higher oil yields but can compromise oilcake quality, while the second can lead to the liberation of aroma compounds and dark colors [90].

In conclusion, the cold oil pressing by-products are characterized by high nutritional values and good functional parameters. Press-cakes can be used as a food ingredient or for the extraction of bioactive compounds that can be incorporated in new foodstuffs because they are nutritional, social and economically advantageous [91].

Oilcakes rich in proteins and lipids are suitable for feeding omnivores and fish while being rich in fibers for ruminants. Studies showed that sunflower oilcakes improved the carcass yield of fish and pigs [92,93].

A possible valorization of sunflower seeds involves the realization of new food products such as tablets that can be used as supplements [70] Other products obtained with sunflower oilcakes were biscuits (higher protein, phenols and antioxidants compounds) [85], cookies (addition of 10% results in better proteins digestibility and water absorption) and muffins (products with low carbohydrate content) [14].

Furthermore, proteins extracted from sunflower oilcakes can be used for the production of films with good adhesive and barrier properties and low elongation, deformation and elasticity [9].

### 2.5. Comparison of the Mineral Composition of the Sunflower Seeds, Oil and Oilcake

Minerals are inorganic nutrients essential for the maintenance of life physicochemical processes [94]. They can be classified into macro-elements (potassium, phosphorus, calcium, sodium and chloride) and micro-elements (iron, copper, zinc, molybdenum, chromium, manganese, copper and selenium). The required amounts in diets for macro-elements must be greater than 100 mg/dL and less for micro-elements [95].

Sunflower seeds, oil and meal are known to be a source of several minerals [10,11,19,34]. The mineral composition is shown in Table 5. A total of 18 elements were found in seeds (Mg < Se < Ce < Ca < Tl < Zn < Mn < Cr < Cu < Ni < Be < Co < Ti < Fe < Li < Mo < Cd) and SFOC/C (Se < Ce < Ca < Tl < Zn < Cu < Mn < Cr < Sr < Be < Ni < Co < Fe < Mo < Li < Cd). In SFOC/PE were found 20 elements as follows: magnesium (4.76 g/Kg), selenium (1.99 g/Kg), cesium (1.02 g/Kg), calcium (1163.32 mg/Kg), thallium (587.97 mg/Kg), zinc (94.78 mg/Kg), strontium (72.97 mg/Kg), copper (61.15 mg/Kg), manganese (57.62 mg/Kg), chromium (52.79 mg/Kg), beryllium (32.96 mg/Kg), nickel (29.38 mg/Kg), titan (16,10 mg/Kg), cobalt (7.63 mg/Kg), iron II and III (5.26 mg/Kg, 3.35 mg/Kg), molybdenum (0.43 mg/Kg), lithium (0.34 mg/Kg), cadmium (0.23 mg/Kg) and antimony (0.02 mg/Kg). They were found only 14 elements in SFO of which thallium, cesium, magnesium and selenium presented high values, the others (Mo < Mn < Be < Cr < Cu < Li < Ni < Zn < Ti < Fe) presented proportions below 1%.

After oil extraction, most mineral composition of oilcakes increased, while Fe, Co and Li decreased. A low percentage of the elements goes into the oil. A comparison between the whole seeds, press-cakes and oil regarding the mineral composition reveals that press-cakes are richer and they are a valuable ingredient for new food products development. The results obtained were in accordance with those provided by other authors [53,96].

Calcium, cobalt, strontium, cadmium and antimony were the only elements that were not found in the oil, despite being present in sunflower seeds and oil. The elements presented only in sunflower oilcakes were strontium and antimony. The difference in elements composition was significant (95% confidence level) for all the samples studied.

### 2.6. Fatty Acids Profile of Sunflower Seeds, Oil and Oilcakes

Fatty acids (FA) composition of the seeds, cakes and oil are shown in Table 6. They were quantified using a gas chromatograph coupled with mass spectrometry. The difference between the seeds, oil and oilcakes was significant (*p* < 0.5%). A total of 14 fatty acids were determined, of which five were saturated (SFA), five monounsaturated (MUFA) and four polyunsaturated (PUFA). The total concentration of FA in seeds and oil were 440.62 µg/mL and 441.31 µg/mL, respectively, while in press-cakes were between 1016.52 µg/mL and 3083.38 µg/mL. The sunflower seeds and oil were rich in PUFA (51.41% and 64.81% respectively) and MUFA (41.69% and 20.58%) and poor in saturated FA (6.90% and 14.61%). The two oilcakes were a rich source of unsaturated FA, namely 29.46% and 57.96% monounsaturated, also 66.13% and 34.16% polyunsaturated. The most abundant FA were linoleic, pentadecanoic, stearic and oleic. Low levels were detected for palmitic, palmitoleic, heptadecenoic, linolelaidic, linolenic, eicosenoic, arachidonic and trisanoic fatty acids.

Palmitic and stearic acid values varied between 2.18% and 5.52%, also between 1.10% and 10.45%, respectively. Values below 1% were found for myristic, linolelaidic, linolenic, eicosenoic, trisanoic and arachidonic fatty acids. The major FA in seeds, cakes and oil were linoleic (50.32%, 32.81–65.88% and 64.35%, respectively) and oleic (14.10%, 10.34–19.32% and 19.81%, respectively). These results make the three products (seeds, oil and oilcakes) important dietary sources of linoleic and oleic fatty acids.

Linolenic and linoleic acids are polyunsaturated essential fatty acids. They can not be synthesized by the organism and play an important role in the maintenance of healthy triglyceride and cholesterol levels. Sunflower linoleic/linolenic FA ratios were high due to the low levels of linolenic acid. Regarding the SFA, the content was relatively low (≤ 14.61%). The consumption of sunflower oil, extracted or naturally presented in seeds or press-cakes, can help to increase the level of linoleic acid in the human body.

Sunflower seeds in the literature presented 9.63–10.11% saturated fatty acids, 20.73–25.77% monounsaturated fatty acids and 65.59–69.64% polyunsaturated fatty acids [59]. In sunflower oil, myristic (<0.2%), palmitic (5–7.6%), palmitoleic (<0.3%), oleic (14.1–39.4%), linoleic (48.3–74%), linolenic (<0.3%) and eicosenoic (<0.5%) were found. All the results are in accordance with those obtained in our study [59].

Values for sunflower meals found in the literature ranged between 11.3%–67.82% for SFA, between 20.6%–25.90% for MUFA and between 3.81–68.2% for PUFA [13]. Regarding the C18:2 w-6/C18:3 w-3 ratio found in our study was in accordance with the values found in the literature (3.86–37.79) [97]. The fatty acids profile in the literature include myristic (0.30%–9.63%), palmitic (12.05–29.1%), stearic (12.2%), linolelaidic (0.04%), linoleic (1.93–57.82%), linolenic (0.39–1.53%), eicosenoic (0.04%), arachidonic (0.02%) and trisanoic (0.05%) fatty acids [98]. Values were in accordance with the results obtained in our study.

In conclusion, sunflower oilcakes can be used for the development of new food products due to their advantageous FA profile, where oleic acid is predominant.

### 2.7. Amino Acids Profile of Sunflower Seeds, Oil and Oilcakes 

Proteins presented the highest increase in press-cakes. To evaluate their quality the amino acids (AA) profile must be determined (Table 7).

The findings showed higher total amino acids content in oilcakes than seeds, 28438.27 nmolg^−1^, 19031.34 nmolg^−1^ and 5790.26 nmolg^−1,^ respectively. In descending order, the amino acids identified in seeds were alanine, glycine, glutamic acid, leucine and aspartic acid. Asparagine and glutamine were not found because they were totally converted to aspartic and glutamic acids in acidic hydrolysis conditions.

In pellets press-cake were found the following 13 AA: valine, glutamic acid, aspartic acid, glycine, alanine, serine, isoleucine, proline, asparagine, threonine, phenylalanine, methionine and leucine. On the other hand, in the cake meal were found 17 AA namely, alanine < glutamic acid < aspartic acid < glycine < serine < tryptophan < valine < proline < isoleucine < phenylalanine < threonine < hydroxylysine < α-aminoadipic acid <methionine < tyrosine < asparagine < leucine.

In cakes, essential AA represents 45.82% and 28.48% of the total AA profile, while in seeds only 6.63%. Valine and tryptophan were the major essential AA found in meals. They were followed by isoleucine, threonine and phenylalanine. All the essential AA must be obtained through an equilibrate diet because they cannot be synthesized in the human body.

Sunflower press cake in the form of pellets presented the most amino acids (28438.27 nmol/g), the difference between all the products was significant (*p* < 95%).

**Figure 1:**
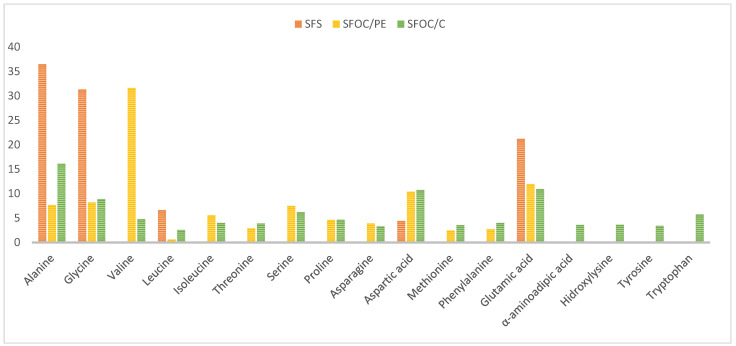
Amino acids of sunflower seeds, meals and oil.

For all the amino acids found in the samples were calculated the percentage relative level which is shown in Figure 1. Based on the relative percentages were calculated the total percentage of essential and non-essential AA (Table 7).

Alanine (difference was significant), glycine (difference was not significant) valine (significant difference) and glutamic acid (significant difference) were the most predominant amino acids, while leucine was the least (significant difference). A total of twelve amino acids (valine, isoleucine, threonine, serine, proline, asparagine, methionine, phenylalanine, α-aminoadipic acid, hidroxylysine, tyrosine and tryptophan) were found in meals but not in the seeds. This is due to the presence of the hulls, which create an intercellular skeleton that prevents the action of digestive enzymes and thus reduces the amino acids present in the hull [99].

## 3. Materials and Methods

### 3.1. Samples

**Figure 2:**
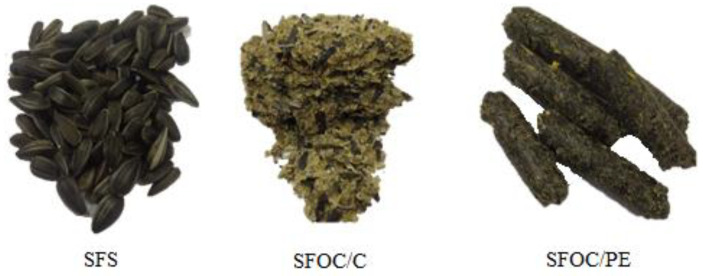
Sunflower seeds and press-cakes.

For this study, high oleic sunflower seeds (*Helianthus annuus L.*), oilcakes and oil were a kind gift from a local factory of Suceava (Romania), that uses traditional mechanical oil pressing technology. The seeds (SFS) (Figure 2) were cleaned manually to remove foreign materials and immature seeds. Kernels and hulls were obtained by manually dehulling.

The two oilcakes have different forms: pellets (SFOC/PE) and cake (SFOC/C) (Figure 2). They were ground and sieved (< 500 µm) with a Retsch Vibratory Sieve Shaker AS 200 basic (Retsch GmbH, Haan, Germany).

### 3.2. Chemical Composition

#### 3.2.1. Seeds and Kernels

Moisture content was measured using a gravimetric method (ISO 665:2020) by drying a 3 g sample in the laboratory oven with air circulation ZRD-A5055 (Zhicheng Analysis Instruments, Shanghai, China) at 105 ± 2 °C for 24 h.

Ash content was estimated with AOAC method 923.03 using a calcination furnace at 550 °C for 6 h till the charred material became white [74].

Protein content was determined using the Kjeldahl method (AOAC 950.48) described by Sunil, 2015 [74] with some modifications and the conversion factor 5.88. Digestion was made as follows: 1 g of sample with 15 mL of sulfuric acid and a catalyst were placed in a Kjeldahl flask. Digestion was carried out at 420 °C for 2 h until a clear blue solution was obtained. Afterward, 50 mL of distilled water is added to the flask. The distillation was carried out with boric acid, while titration with hydrochloric acid was 0.2 N.

The lipid content of seeds, kernels and hulls was determined by ISO 659:2009 using an automatic Soxhlet extraction system with n-hexane solvent.

#### 3.2.2. Oilcakes

The oilcakes were investigated for their moisture, ash, fat, protein and total dietary fiber (Megazyme total dietary fiber assay kit, Megazyme, Wicklow, Ireland) with AOAC methods with some modifications (methods 935.29, 923.03, 920.39, 950.48 and 985.29 respectively).

The color of the press-cakes was measured using a CR-400 colorimeter (Konica Minolta, Tokyo, Japan) and CIELAB scale: lightness L* (0 for black and 100 for white), a* (if negative indicate the intensity of green, if positive of red) and b*(if negative indicate the intensity of blue, if positive of yellow) [100].

The water/oil holding capacity (WHC, OHC) was measured according to the method described by Omowaye-Taiwo, 2015 [101], with slight modifications. In test tubes, 1 g of sample and 10 mL of distilled water/corn oil were added. Then they were kept at room temperature for 30 min and centrifugated at 7000 rpm for 20 min. The results were expressed as grams of water/oil absorbed per gram of sample.

Water retention capacity (WRC) was determined according to the method described by Onipide et al., 2017 [102]. The sample (2 g) was mixed with 20 mL distilled water, kept for 1 h at 25 °C in a water bath (Memmert Waterbath WNB 22, Schwabach Germany) with continuous stirring and centrifugated at 1600 rpm for 25 min. The supernatant was removed and the samples were weighed. WRC was calculated as the difference between the hydrated and dry residues.

To measure the swelling capacity (SC), 1 g of sample and 10 mL of distilled water were mixed in a centrifuge tube. After centrifugation for 20 min at 2000 rpm, the supernatant was decanted and the remained sample was dried in a hot air oven for 2 h at 130 °C and then weighted. The results were expressed as percent swelled per gram sample [103].

Bulk density (BD) of sunflower meals was analyzed by a volumetric method; 5 g of sample were placed in a 100 mL cylinder and gently tapped 20 times. The values were calculated as sample weight and volume displaced ratio (g/mL) [80].

Emulsion capacity (EC) and stability (ES) were analyzed according to Rani, 2021 [72] and Yyenagbe et al., 2017 [104] as follows: a 0.5% suspension of sample and water was mixed on a magnetic stirrer for 20 min at 500 rpm, 30 mL of this emulsion were then taken and mixed with corn oil (10 mL). The emulsion was homogenized and transferred immediately to a marked cylinder (50 mL) in order to read the height obtained. The ES was calculated by heating the cylinder for 30 min at 80 °C. After that, the final height of the emulsion was read. The formulas for calculating EC and ES are expressed in Equations (1) and (2).

(1) $$EC\;\left(\%\right)\;=\;\frac{\mathrm{Height}\;\mathrm{of}\;\mathrm{oil}\;\mathrm{layer}}{\mathrm{Height}\;\mathrm{of}\;\mathrm{the}\;\mathrm{suspension}}\;\times \;100$$

(2) $$ES\;\left(\%\right)\;=\;\frac{\mathrm{Final}\;\mathrm{height}\;\mathrm{of}\;\mathrm{the}\;\mathrm{emulsion}\;\mathrm{layer}}{\mathrm{Initial}\;\mathrm{height}\;\mathrm{of}\;\mathrm{the}\;\mathrm{emulsion}\;\mathrm{layer}}\;\times \;100$$

### 3.3. Physical Properties of Seeds and Kernels

#### 3.3.1. Mass and Classification

A group of randomized whole seeds was weighted with an analytical balance PARTNER AS 220.R2 (Radwag, Bucharest, Romania) at 0.1 mg accuracy. In this way, the percentage of hull and kernel was determined. Moreover, based on mass, the kernels and seeds were classified into three categories [40].3.3.2. Dimensional Parameters

**Figure 3:**
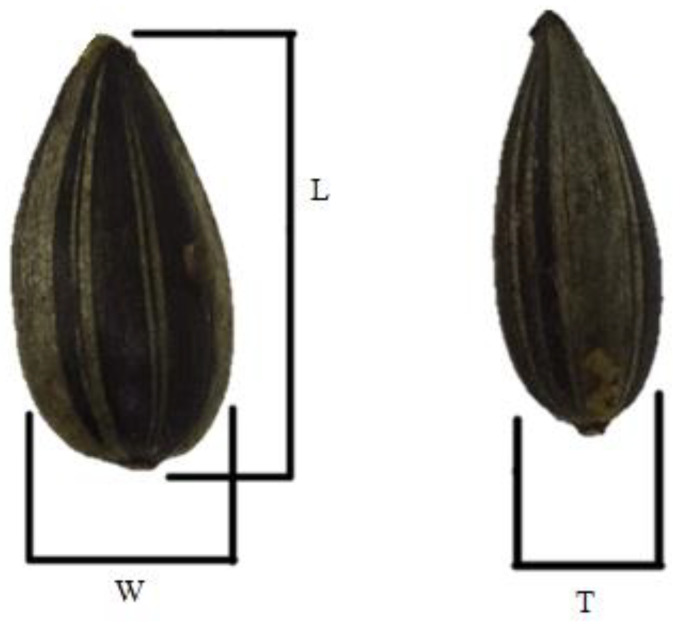
Spatial dimension of sunflower seeds; L—length, W—width, T—thickness.

Groups of 100 seeds were randomly measured to determine their dimensions. Length (L), width (W) and thickness (T) were analyzed using a digital caliper VOREL 15240 (Toya, Wrocław, Poland) at 0.003 mm accuracy [40]. In the same way, the size and shape (l, w and t) of kernels were determined (Figure 3).

#### 3.3.2. Geometric Parameters

The equivalent diameter (D_e_), sphericity (ψ), seed surface area (S), projected area (A_p_) and volume (V) were determined by comparison to a sphere using the following relationships [37,39,40,49]:

(3) $$\mathrm{De}\;(\mathrm{mm})\;=\;\sqrt[{3}]{L\;\times \;W\;\times \;T}$$

(4) $$\psi \;=\;D_{e}/L$$

(5) $$S\;({\mathrm{mm}}^{2})\;=\;𝛱\;\times \;{D_{e}}^{2}$$

(6) $$A_{p}\;({\mathrm{mm}}^{2})\;=\;¾\times L\;\times \;W$$

(7) $$V({\mathrm{mm}}^{3})\;=\;W\;\times \;L\;\times \;T\;\times \;\varphi$$

#### 3.3.3. Gravimetric Parameters

Bulk density (p_b_) was calculated as the ratio of the seeds/kernels mass (M/m) and their total occupied volume (V). The method described by Konak et al., 2002 [105], with some modifications, was used.

p_b_ (kg/m^3^) = M/V (8)

True density (p_t_) was analyzed with toluene displacement method using a pycnometer. The method consists in immersion of a weighted quantity of seeds in toluene (*p* = 0.867 g/mL) and then recording the volume displaced [40].

(9) $$p_{t}\;(\mathrm{kg}/m^{3})\;=\;(M\;\times \;p_{\mathrm{toluene}})/M_{T}$$

Porosity was calculated as a function of the previous densities according on the Equation (10) [37,39,40,49].

(10) $$pt_{\;}(\%)\;=\;\frac{\mathrm{pt}-\;\mathrm{pb}}{\mathrm{pt}}\;\times 100$$

### 3.4. Comparison of the Seeds, Oil and Oilcakes

#### 3.4.1. Free Amino Acids Determination

Prior to analysis, the sample (3.7 ± 0.5 g) was mixed with 30 mL of 15% trichloroacetic acid (TCA). The pH of the solution was adjusted to 2.2 with sodium hydroxide solution and further diluted to exactly 50 mL with 15% TCA. After centrifugation for 5 min at 3000 rpm, the supernatant was filtrated through a 0.45 µm nylon filter [106,107]. The solution contains primary and secondary amino acids that were further analyzed with the Ez:faast GC-MS kit (Phenomenex, Torrance, CA, USA). The method was carried out in three steps: a solid phase extraction (performed in sorbent packed tips that bids amino acids and allow the other compounds to flow through), quick derivatization (amino acids migrate to the organic layer) and a liquid/liquid extraction (the organic layer was removed, evaporated and re-dissolved in solvent). Amino acids analysis was made with a gas-chromatograph coupled with a mass spectroscopy instrument (Shimadzu, Kyoto, Japan). The entire time of analysis was 10 min and the injected volume was set at 0.002 mL. The amino acids separation was performed in a ZB-AAA (10 m × 0.25 mm) column. It was applied the split-less injection mode. The initial temperature of the GC oven was 110 °C, which was increased until 320 °C and held for three minutes. The condition employed for the mass spectrometer were 200 °C for the ion source and 320 °C for the interface. The quadrupole measured the abundance of ions from 35 to 500 m/z. For the calibration, there were used solutions with amino acids mixture included in the kit mentioned above [107].

#### 3.4.2. Fatty Acids Determination

The oils extracted from oilcakes and seeds and the one provided by the local factory were analyzed for fatty acids methyl esters (FAME). Fatty acids derivation was performed according to the following procedure: 0.1 g of each oil sample was mixed with 0.4 mL of n-hexane and 0.4 mL of 15% boron trifluoride in methanol. The solution was heated at 60 °C in a water bath (Memmert Waterbath WNB 22, Schwabach, Germany) for 5 min. After cooling to room temperature, it was mixed with 2 mL of saturated sodium chloride solution and centrifugated at 2000 rpm for three minutes. After that, the supernatant was filtrated through a 0.45 µm nylon filter. FAME’s separation was made on a GC-MS instrument (GC MS-QP 2010 Plus, Shimadzu, Kyoto, Japan), using a SUPELCOWAX 10 column (length of 60 m, inner diameter of 0.25 mm ID and 0.25 µm film thickness; Supelco Inc., Bellefonte, PA, USA). The GC oven temperature increased at a rate of 7 °C/min from 140 °C to 220 °C, which was held for 23 min. The flow rate of the carrier gas (He) was kept at 0.8 mLmin^−1^. The injection port temperature was 210 °C. The temperature condition employed for the mass spectrometer were 250 °C for interface and 180 °C for the ion source. Ion electron impact mass spectra were recorded at a positive ionization energy of 70 eV. The scans range was 22–395 m/z (0.14 scans/s). Under these conditions, a 0.001 mL sample was injected in split mode 1:24. FAME’s identification and quantification were performed by comparing their retention times with calibration curves obtained by reference standard FAME’s mixed (Restek, Lisses, France) [108].

#### 3.4.3. Mineral Estimation

The mineral elements were estimated with a system Agilent Technologies 7500 Series (Agilent Technologies, Santa Clara, CA, USA) coupled-mass spectrometer. The parameters were nebulizer 0.9 mL/min, RF power 1500 W, carrier gas 0.92 L/min, makeup gas 0.17 L/min, mass range 7–205 uma, integration time 0.1 s, acquisition 22.76 s. Detector parameters were: discriminator 8 mV, analogue HV 1770 V and pulse HV 1070 V. Prior to analysis, five grams of sample were calcinated at 550˚C for 6 h. The ash was dissolved with 0.73 mL nitric acid (65% HNO3, Sigma Aldrich, Darmstadt, Germany), placed into a 50 mL flask and completed with deionized water. The elements standard solutions were prepared by diluting stock solution of 1000 mg/L of Li, Be, Mg, Ti, Tl, Co, As, Ca, Cd, Cr, Ce, Cu, Hg, Fe, Mn, Ni, Pb, Se, Sr, Sb, Mo, V and Zn [73].

### 3.5. Statistical Analysis

All results are presented as mean ± standard deviation. The chemical and functional analyses for sunflower seeds and oilcakes were performed in triplicate. While, amino acids, fatty acids and minerals analyses were performed in duplicate. The physical characteristics of sunflower seeds were performed on a sample of 500 seeds. The values obtained were processed by using SPSS 25.0 (trial version) software (IBM, New York, NY, USA). The difference between samples was established by analysis of variance (ANOVA) using Turkey’s test at a 5% significance level. To determine the difference between the two samples of oilcakes a Student’s *t*-test was performed.

## 4. Challenges and Future Perspective

Sunflower oilcakes, the by-products obtained after the extraction of oil for sunflower seeds, are rich in dietary fibers and proteins. In the literature, we found numerous studies about the extraction of proteins and their utilization in the food industry, but none about the utilization of dietary fibers. Moreover, there is an increasing demand for an alternative source of plant proteins opening an opportunity for the utilization of sunflower oilcakes as a source of proteins, due to the well-balanced amino acid profile.

Sunflower oilcakes represent also a successful alternative for future food packaging because they are renewable, low-cost resources. Moreover, they permit the development of completely edible and biodegradable materials.

## 5. Conclusions

Nowadays consumers are interested in healthy lifestyles with diets rich in nutrients and for this reason, the consumption of sunflower seeds, with a high nutritive profile, is recommended. Moreover, the current environmental issues caused by the large amount of waste produced by the food industry impose the research of new ways to transform and re-circulate these resources (very rich in bioactive compounds) into the supply chain.

For increasing the interest in food products containing valuable compounds it is necessary to reduce the prices and improve sensorial acceptance. In this sense, they have evaluated the physicochemical and functional properties of sunflower oilcake. The conclusion obtained indicated that sunflower oilcakes represent due to their nutritive properties a good source for good functional products, conferring also good sensorial characteristics.

The high composition in essential amino acids (45.82% for SFOC/PE and 28.4% for SFOC/C), fibers (31.88% and 12.64% for the oilcakes in form of pellets and cake, respectively) and proteins (21.60% for SFOC/C and 20.15 for SFOC/PE) make the oilcakes an interesting option for human consumption. Moreover, they still contain lipids (15.77% for pellets and 14.16% for cake) rich in unsaturated fatty acids (95.59% and 92.12% for pellets and cake, respectively). The predominant fatty acids are oleic (in pellets 29.32% and in cake 10.34%) and linoleic (32.81 and 65.88% for cake and pellets, respectively) acids which are essential because they cannot be synthesized in the human body. The amino acids profile shows a balanced composition between essential and non-essential (SFOC/PE presents 45.82% and 54.18% essential and non-essential AA respectively). The valorization of these by-products by incorporation in the human diet contributes to the realization of sustainability and circular economy.

Sunflower seeds are very nutritive (33.85% proteins, 65.42% lipids and 18 mineral elements). Due to the rich content in lipids, they are principally used as a source for vegetable oil, which is predominant in kernels (23.73%). To choose high-quality sunflower seeds with the highest fat contents, a classification based on the mass is required. An attentive analysis shows that class III permits obtaining a high oil yield. Further analysis of the extracted oil shows high content in unsaturated fatty acids (93.1%), of which the main are oleic and linoleic acids (respectively, 14.10% and 50.32%). The seeds are a rich source (the value for total AA present in the sample is 5790.26 nmol/g) of essential (6.63%) and non-essential (93.37%) amino acids. On the other hand, the oil extracted from oilcakes and that obtained by cold-pressing present similar chemical properties. Sunflower oil presents essential fatty acids, namely 19.81% oleic acid and 64.35% linoleic acid. Together they help reduce cholesterol levels in the blood and thus prevent heart diseases. A nutritional comparison between oilcakes, seeds and oil shows that they have numerous components suitable for a healthy diet.

## Figures and Tables

**Table 1:** Chemical composition of the whole seeds, kernels and hulls.

Sample	Moisture, %	Ash, %	Proteins, %	Lipids, %
Seed	6.16 ± 0.04 ^b^	2.73 ± 0.04 ^b^	33.85 ± 0.88 ^b^	65.42 ± 0.4 ^a^
Kernel	4.60 ± 0.03 ^c^	3.31 ± 0.11 ^a^	23.73 ± 1.31 ^a^	32.50 ± 2.21 ^b^
Hull	7.88 ± 0.09 ^a^	2.45 ± 0.11 ^c^	7.82 ± 0.22 ^c^	8.81 ± 0.12 ^c^

Different superscripts letters after the values indicated differences statistically significant at *p* < 0.05%.

**Table 2:** Correlation between the dimensional and gravimetric properties of sunflower seeds and kernels, *n* = 145.

Parameters	Ratio Value	Correlation Coefficient
L/W	2.04	0.693 *
L/T	3.34	0.567 *
W/T	1.64	0.828 *
l/w	2.17	0.431 *
l/t	3.65	0.828 *
w/t	1.68	0.404 *
L/M	182.65	0.625 *
W/M	89.69	0.805 *
T/M	54.67	0.793 *
D_e_/M	96.24	0.841 *
ψ/M	8.67	0.615 *
l/m	183.77	0.599 *
t/m	50.43	0.511 *
w/m	84.81	0.626 *
D_ek_/m	9.21	0.723 *
Ψ_k_/m	1.07	0.259 *
L/m	23.91	0.617 *
W/m	11.74	0.809 *
T/m	7.16	0.791 *
D_e_/m	12.60	0.840 *
ψ/m	1.14	0.623 *
M/m	1.31	0.965 *
L/w	2.82	0.538 *
L/l	1.30	0.453 *
W/w	1.39	0.679 *
T/t	1.42	0.603 *

The symbol * indicates significance at *p* < 0.01; L—length of the whole seeds; W—width of the whole seeds; T—thickness of the whole seeds; M—mass of the whole seeds; l, w, t, m—length, width, thickness and mass of the kernels; D_e_—equivalent diameter of the sunflower seeds; D_ek-_—equivalent diameter of sunflower kernels; ψ, ψ_k_—sphericity of the sunflower seeds and kernels, respectively.

**Table 3:** Dimensional and gravimetric parameters for the unsorted seeds and kernels and their three categories, *n* = 500.

Type	Parameters	Unsorted	Classification Based on Mass		
			Class I	Class II	Class III
Shape and spatial dimension					
Seed	L, mm	11.16 ± 0.03 ^b^	10.27 ± 0.74 ^d^	10.83 ± 0.71 ^c^	12.20 ± 0.53 ^a^
	W, mm	5.48 ± 0.02 ^b^	4.13 ± 0.10 ^d^	5.25 ± 0.29 ^c^	6.53 ± 0.29 ^a^
	T, mm	3.34 ± 0.01 ^b^	2.42 ± 0.26 ^d^	3.21 ± 0.24 ^c^	4.01 ± 0.25 ^a^
	D_e_, mm	5.88 ± 0.07 ^b^	4.67 ± 0.18 ^d^	5.66 ± 0.19 ^c^	6.83 ± 0.19 ^a^
	Ψ, -	0.53 ± 0.01 ^b^	0.46 ± 0.03 ^c^	0.52 ± 0.03 ^b^	0.56 ± 0.02 ^a^
	V, mm^3^	113.88 ± 5.12 ^b^	46.83 ± 3.53 ^d^	95.60 ± 5.26 ^c^	178.80 ± 6.94 ^a^
	S, mm^2^	110.15 ± 2.54 ^b^	68.59 ± 5.16 ^d^	100.84 ± 6.11 ^c^	146.47 ± 8.27 ^a^
	A_p_, mm^2^	48.48 ± 0.89 ^b^	33.33 ± 2.56 ^d^	44.65 ± 3.71 ^c^	62.56 ± 4.49 ^a^
Kernel	L, mm	8.58 ± 0.63 ^b^	7.52 ± 0.48 ^c^	8.55 ± 0.51 ^b^	9.15 ± 0.41 ^a^
	W, mm	3.96 ± 0.25 ^b^	3.47 ± 0.21 ^c^	3.8 ± 0.28 ^b^	4.44 ± 0.28 ^a^
	T, mm	2.35 ± 0.16 ^b^	2.05 ± 0.18 ^c^	2.22 ± 0.17 ^b^	2.69 ± 0.19 ^a^
	D_ek_, mm	4.30 ± 0.13 ^b^	3.8 ± 0.15 ^d^	4.15 ± 0.11 ^c^	4.78 ± 0.12 ^a^
	Ψ_k_, -	0.50 ± 0.03 ^b^	0.50 ± 0.03 ^b^	0.49 ± 0.03 ^b^	0.52 ± 0.02 ^a^
	V_k_, mm^3^	41.05 ± 3.56 ^b^	26.99 ± 1.15 ^d^	34.99 ± 3.31 ^c^	57.02 ± 4.94 ^a^
	S_k_, mm^2^	58.39 ± 3.61 ^b^	44.37 ± 3.8 ^d^	54.20 ± 2.86 ^c^	71.66 ± 3.57 ^a^
	A_pk_, mm^2^	26.76 ± 2.35 ^b^	20.49 ± 1.51 ^c^	25.50 ± 2.18 ^b^	31.88 ± 2.60 ^a^
Gravimetric properties					
Seed	M, g	0.0611 ± 0.002 ^b^	0.0395 ± 0.001 ^c^	0.0569 ± 0.003 ^b^	0.07856 ± 0.007 ^a^
	p_b_, Kg/m^3^	404.54 ± 2.76 ^b^	395.23 ± 2.53 ^c^	415.08 ± 2.49 ^b^	425.47 ± 3.13 ^a^
	p_t_, Kg/m^3^	704.81 ± 1.15 ^a^	708.07 ± 4.63 ^a^	691.22 ± 1.3 ^b^	650.33 ± 2.25 ^c^
	φ, Kg/m^3^	42.60 ± 0.77 ^b^	44.18 ± 0.95 ^a^	39.95 ± 0.93 ^c^	34.58± 0.18 ^d^
Kernel	m, g	0.0467 ± 0.003 ^b^	0.0292 ± 0.002 ^c^	0.0403 ± 0.003 ^b^	0.0591 ± 0.004 ^a^
	p_b_, Kg/m^3^	525.29 ± 4.03 ^b^	414.81 ± 5.29 ^d^	484.00 ± 3.05 ^c^	598.08 ± 4.43 ^a^
	p_t_, Kg/m^3^	1072.13 ± 0.75 ^b^	1079.69 ± 0.45 ^a^	1074.41 ± 1.21 ^c^	1068.60 ± 0.73 ^d^
	φ, Kg/m^3^	51.02 ± 0.03 ^c^	61.58 ± 0.02 ^a^	54.95 ± 0.05 ^b^	44.03 ± 0.04 ^d^

L, W, T, M, D_e_, ψ, V, S, A_p_—length, width, thickness, mass, equivalent diameter, sphericity, volume, surface area and projected area of sunflower seeds; l, w, t, m, D_ek_, ψ_k_, V_k_, S_k_, A_pk_—length, width, thickness, mass, equivalent diameter, sphericity, volume, surface area and projected area of sunflower kernels; p_b_—bulk density; p_t_—true density; φ—porosity. Different superscript letters indicated difference at *p* < 0.05%.

**Table 4:** Chemical, functional and color proprieties of the two sunflower oilcakes.

Parameters	SFOC/PE	SFOC/C
PHYSICO-CHEMICAL PROPERTIES		
Moisture (%)	8.75 ± 0.10 ^a^	8.93 ± 0.11 ^a^
Dry matter (%)	91.25 ± 0.10 ^a^	91.07 ± 0.11 ^a^
Proteins (%)	20.15 ± 1.57 ^a^	21.60 ± 1.87 ^a^
Fat (%)	15.77 ± 0.45 ^a^	14.16 ± 0.04 ^b^
Ash (%)	4.56 ± 0.11 ^b^	6.15 ± 0.04 ^a^
Crude fiber (%)	31.88 ± 0.79 ^a^	12.64 ± 0.05 ^b^
Carbohydrates (%)	18.89 ± 0.23 ^a^	36.52 ± 1.11 ^b^
FUNCTIONAL PROPERTIES		
Bulk density (g/mL)	0.4196 ± 0.002 ^a^	0.4204 ± 0.001 ^a^
WHC (g/g)	2.58 ± 0.11 ^a^	2.33 ± 0.07 ^b^
OHC (g/g)	1.34 ± 0.13 ^a^	1.18 ± 0.08 ^a^
WRC (g/g)	4.67 ± 0.04 ^a^	5.51 ± 0.06 ^a^
SC (%)	3.56 ± 0.06 ^a^	3.19 ± 0.17 ^a^
EC (%)	32.17 ± 1.15 ^a^	30.62 ± 2.14 ^a^
ES (%)	29.87 ± 1.24 ^a^	27.92 ± 0.57 ^a^
COLOUR PROPERTIES		
L*	42.23 ± 0.01 ^b^	46.29 ± 0.01 ^a^
a*	1.17 ± 0.01 ^b^	1.57 ± 0.01 ^a^
b*	6.11 ± 0.01 ^b^	8.90 ± 0.01 ^a^

SFOC/PE—sunflower oilcakes in pellets form; SFOC/C—sunflower oilcake in the form of cake; WHC—water holding capacity; OHC—oil holding capacity; WRC—water retention capacity; SC—swelling capacity, EC—emulsion capacity; ES—emulsion stability; L*—lightness; a*—redness; b*—yellowness. For difference assessment was performed Student t-test. Values followed by ^a^, ^b^ are statistically different at 95% confidence level.

**Table 5:** Mineral composition of the sunflower seeds, oil and press-cakes.

Parameters	SFS mg/Kg	SFOC/PE mg/Kg	SFOC/C mg/Kg	SFO mg/Kg
Li	1.80 ± 0.01 ^a^	0.34 ± 0.01 ^c^	1.40 ± 0.0 ^b^	0.20 ± 0.01 ^c^
Be	20.89 ± 0.14 ^c^	32.96 ± 0.55 ^a^	31.48 ± 0.06 ^b^	0.72 ± 0.03 ^d^
Mg	3.89 ± 0.24^1 b^	4.76 ± 0.13^1 a^	-	3.44 ± 0.15 ^c^
Ca	573.02 ± 4.73 ^c^	1163.32 ± 10.01 ^b^	1522.08 ± 5.5 ^a^	-
Ti	7.04 ± 0.25 ^c^	16.10 ± 0.26 ^b^	18.38 ± 6.22 ^a^	0.03 ± 0.0 ^d^
Cr	35.70 ± 0.1 ^c^	52.79 ± 0.38 ^b^	58.24 ± 1.81 ^a^	0.50 ± 0.01 ^d^
Mn	36.91 ± 0.38 ^c^	57.62 ± 0.21 ^b^	65.73 ± 3.46 ^a^	0.78 ± 0.03 ^d^
Fe (II)	6.66 ± 0.13 ^a^	5.26 ± 0.20 ^b^	4.71 ± 2.71 ^c^	0.01 ± 0. ^d^
Fe (III)	6.40 ± 0.44 ^a^	3.35 ± 0.17 ^b^	2.51 ± 0.03 ^c^	-
Co	11.46 ± 0.69 ^a^	7.63 ± 0.41 ^b^	5.59 ± 0.35 ^c^	-
Ni	21.29 ± 1.30 ^c^	29.38 ± 1.27 ^b^	30.63 ± 1.40 ^a^	0.19 ± 0.01 ^d^
Cu	32.57 ± 1.79 ^c^	61.15 ± 4.12 ^b^	71.25 ± 3.36 ^a^	0.21 ± 0.01 ^d^
Zn	57.83 ± 2.54 ^c^	94.78 ± 2.28 ^b^	90.11 ± 4.36 ^a^	0.09 ± 0.0 ^d^
As	-	-	-	-
Se	1.22 ± 0.02^1 c^	1.99 ± 0.07^1 b^	3.18 ± 0.18^1 a^	1.17 ± 0.0 ^d^
Sr	-	72.97 ± 2.42 ^a^	35.42 ± 1.99 ^b^	-
Mo	0.34 ± 0.0 ^c^	0.43 ± 0.01 ^c^	1.62 ± 0.55 ^a^	0.90 ± 0.01 ^b^
Cd	0.16 ± 0.00 ^c^	0.23 ± 0.01 ^b^	0.26 ± 0.0 ^a^	-
Sb	-	0.02 ± 0.0 ^a^	-	-
Ce	0.33 ± 0.01 ^1 d^	1.02 ± 0.01 ^1 b^	1.85 ± 0.05 ^1 a^	6.39 ± 0.35 ^c^
Tl	523.84 ± 9.11 ^b^	587.97 ± 17.80 ^a^	417.12 ± 7.31 ^d^	175.69 ± 4.56 ^c^

The superscript number ^1^ indicates that the results are expressed as g/Kg. The analysis was performed in duplicate. Results are presented as values ± standard deviation. SFS—sunflower seeds, SFOC/PE—pellets sunflower oilcakes, SFOC/C—sunflower oilcakes in the form of cake, SFO—sunflower oil, Li—lithium, Be—beryllium, Mg—magnesium, Ca—calcium, Ti—titan, Cr—chromium, Mn—manganese, Fe—iron, Co—cobalt, Ni—nickel, Cu—copper, Zn—zinc, As—arsenic, Se—selenium, Sr—strontium, Mo—molybdenum, Cd—cadmium, Sb—antimony, Ce—cesium, Tl—thallium. Lowercase letters (^a^,^b^,^c^ and ^d^) refer to the comparison of the same element between the different samples; results followed by superscript letters are significantly different (*p* < 0.05%) according to Turkey’s post hoc test.

**Table 6:** Fatty acids composition of sunflower seeds, oil and oilcake.

Fatty Acid ^1^	Type	SFS ^2^ µg/mL	Relative Level ^3^ %	SFOC/PE µg/mL	Relative Level %	SFOC/C µg/mL	Relative Level %	SFO µg/mL	Relative Level %
C14:0	SFA	2.25 ± 0.20 ^c^	0.43 ± 0.02 ^A^	5.05 ± 0.02 ^a^	0.16 ± 0.04 ^C^	3.78 ± 0.02 ^b^	0.31 ± 0.00 ^B^	-	-
C15:1	MUFA	47.98 ± 0.20 ^b^	27.60 ± 0.50 ^B^	-	-	148.28 ± 0.36 ^a^	36.82 ± 0.09 ^A^	-	-
C16:0	SFA	38.46 ± 0.11 ^d^	2.44 ± 0.12 ^C^	210.89 ± 1.35 ^a^	2.18 ± 0.12 ^D^	201.91 ± 0.54 ^b^	5.52 ± 0.32 ^A^	41.07 ± 0.04 ^c^	3.41 ± 0.12 ^B^
C16:1	MUFA	-	-	0.96 ± 0.00 ^c^	0.03 ± 0.00 ^C^	123.01 ± 0.95 ^a^	10.08 ± 0.53 ^A^	3.08 ± 0.07 ^b^	0.77 ± 0.04 ^B^
C17:1	MUFA	-	-	1.03 ± 0.04 ^b^	0.06 ± 0.00 ^B^	4.38 ± 0.02 ^a^	0.72 ± 0.04 ^A^	-	-
C18:0	SFA	26.47 ±0.07 ^c^	2.51 ± 0.06 ^B^	114.30 ± 0.98 ^a^	1.77 ± 0.15 ^C^	26.91 ± 0.18 ^c^	1.10 ± 0.09 ^D^	83.77 ± 0.54 ^b^	10.45 ± 0.34 ^A^
C18:1 (w-9)	MUFA	49.47 ± 0.20 ^d^	14.10 ± 0.32 ^C^	629.87 ± 0.35 ^a^	29.32 ± 0.95 ^A^	84.06 ± 0.54 ^b^	10.34 ± 0.45 ^D^	52.97 ± 0.32 ^c^	19.81 ± 0.54 ^B^
C18:2 (all-trans 9,12) (w-6 t)	PUFA	3.98 ± 0.01 ^b^	0.76 ± 0.03 ^A^	2.90 ± 0.07 ^c^	0.09 ± 0.04 ^C^	8.17 ± 0.03 ^a^	0.67 ± 0.04 ^B^	-	-
C18:2 (all-cis 9,12) (w-6)	PUFA	262.34 ± 0.11 ^c^	50.32 ± 1.49 ^C^	2102.26 ± 5.55 ^a^	65.88 ± 1.55 ^A^	396.30 ± 0.17 ^b^	32.81 ± 1.85 ^D^	255.59 ± 1.32 ^d^	64.35 ± 0.14 ^B^
C18:3 (w-3)	PUFA	1.70 ± 0.02 ^c^	0.33 ± 0.04 ^B^	5.35 ± 0.15 ^a^	0.17 ± 0.00 ^C^	2.17 ± 0.12 ^b^	0.18 ± 0.04 ^C^	1.83 ± 0.42 ^c^	0.46 ± 0.02 ^A^
C20:1 (w-9)	MUFA	-	-	1.25 ±0.04 ^a^	0.04 ± 0.00 ^A^	-	-	-	-
C20:4 (w-6)	PUFA	-	-	-	-	6.07 ± 0.22 ^a^	0.50 ± 0.03 ^A^	-	-
C21:0	SFA	7.99 ± 0.07 ^b^	1.53 ± 0.00 ^A^	6.68 ± 0.98 ^c^	0.21 ± 0.07 ^D^	11.50 ± 0.54 ^a^	0.95 ± 0.04 ^B^	3.00 ± 0.22 ^d^	0.75 ± 0.04 ^C^
C23:0	SFA	-	-	2.86 ± 0.07 ^a^	0.09 ± 0.04 ^A^	-	-	-	-
C18:2 w-6/C18:3 w-3		152.49 ± 0.98 ^C^		387.53 ± 4.55 ^A^		182.28 ± 1.95 ^B^		139.89 ± 0.00 ^D^	
C18:1 w-9/C18:2 w-6		0.28 ± 0.01 ^C^		0.45 ± 0.01 ^A^		0.32 ± 0.001 ^B^		0.31 ± 0.00 ^B^	
ΣSFAs (%)		6.90 ± 0.04 ^C^		4.41 ± 0.09 ^D^		7.88 ± 0.07 ^B^		14.61 ± 0.04 ^A^	
ΣUFAs (%)		93.1 ± 0.54 ^B^		95.59 ± 0.98 ^A^		92.12 ± 1.55 ^C^		85.39 ± 1.49 ^D^	
ΣMUFAs (%)		41.69 ± 1.54 ^B^		29.46 ± 0.54 ^C^		57.96 ± 0.32 ^A^		20.58 ± 0.17 ^D^	
ΣPUFAs (%)		51.41 ± 1.32 ^C^		66.13 ± 0.25 ^A^		34.16 ± 0.15 ^D^		64.81 ± 0.35 ^B^	
ΣSFAs/ΣUFAs		0.07 ± 0.00 ^C^		0.05 ± 0.00 ^C^		0.09 ± 0.00 ^B^		0.17 ± 0.00 ^A^	

^1^ C14:0, Myristic; C15:1, Pentadecenoic; C16:0, Palmitic; C16:1, Palmitoleic; C17:1, Heptadecenoic; C18:0, Stearic; C18:1, Oleic; C18:2 (all trans 9.12), Linolelaidic; C18:2 (all-cis 9.12), Linoleic; C18:3, Linolenic; C20:1, Eicosenoic; C20:4, Arachidonic; C21:0, Heneicosanoic acid; C23:0 Trisanoic acids; SFAs, saturated: UFAs, unsaturated; MUFAs, monosaturated; PUFAs, polyunsaturated fatty acids. ^2^ Values are presented as mean± standard deviation. When followed by different superscript letters (^a^, ^b^, ^c^, ^d^) they are statistically different at 95% confidence level. ^3^ Uppercase superscript refers to relative level %. Values with different letters (^A^, ^B^, ^C^, ^D^) are statistically different at *p* < 0.05%

**Table 7:** Amino acids profile of sunflower seeds, oil and oilcake.

Parameters	SFS nmol/g	SFOC/PE nmol/g	SFOC/C nmol/g
Alanine	2110.4 ± 18.21 ^b^	2187.18 ± 36.93 ^b^	3073.51 ± 43.48 ^a^
Glycine	1810.93 ± 0.0 ^a^	2329.15 ± 0.0 ^a^	1696.04 ± 324.38 ^a^
Valine *	-	8987.78 ± 3.40 ^a^	905.97 ± 22.10 ^b^
Leucine *	383.84 ± 0.0 ^a^	164.77 ± 4.20 ^b^	486.66 ± 8.00 ^a^
Isoleucine *	-	1584.28 ± 14.59 ^a^	757.88 ± 2.04 ^b^
Threonine *	-	827.21 ± 17.53 ^a^	742.32 ± 8.33 ^b^
Serine	-	2124.69 ± 12.66 ^a^	1181.12 ± 26.93 ^b^
Proline	-	1313.13 ± 8.66 ^a^	887.04 ± 12.66 ^b^
Asparagine	-	1102.81 ± 10.90 ^a^	629.15 ± 3.76 ^b^
Aspartic acid	255.53 ± 3.58 ^c^	2949.61 ± 137.36 ^a^	2045.89 ± 19.58 ^b^
Methionine *	-	696.83 ± 3.92 ^a^	675.17 ± 1.90 ^b^
Phenylalanine *	-	768.82 ± 1.56 ^a^	757.25 ± 8.43 ^b^
Glutamic acid	1229.56 ± 0.0 ^a^	3402.01 ± 0.0 ^b^	2082.36 ± 39.83 ^a^
α-aminoadipic acid	-	-	680.19 ± 18.43 ^a^
Hidroxylysine	-	-	686.60 ± 33.33 ^a^
Tyrosine	-	-	650.22 ± 0.32 ^a^
Tryptophan *	-	-	1093.97 ± 14.53 ^a^
Total, nmol	5790.26 ^c^	28438.27 ^a^	19031.34 ^b^
Essential AA, %	6.63 ^c^	45.82 ^a^	28.48 ^b^
Non essential AA, %	93.37 ^a^	54.18 ^c^	71.52 ^b^

The symbol * indicates an essential amino acid. The analysis was performed in duplicate. Values are presented as mean ± standard deviation. Values followed by different superscript letters (^a^, ^b^, ^c^, ^d^) are statistically different at 95% confidence level.

## Data Availability

Not applicable.

## References

[1] Otles S., Despoudi S., Bucatariu C., Kartal C. (2015). Valorization, and Sustainability in the Food Industry.

[2] Kot A.M., Pobiega K., Piwowarek K., Kieliszek M., Błażejak S., Gniewosz M., Lipińska E. (2020). Biotechnological Methods of Management and Utilization of Potato Industry Waste—A Review. Potato Res..

[3] Esposito B., Sessa M.R., Sica D., Malandrino O. (2020). Towards circular economy in the agri-food sector. A systematic literature review. Sustainability.

[4] Borrello M., Caracciolo F., Lombardi A., Pascucci S., Cembalo L. (2017). Consumers’ perspective on circular economy strategy for reducing food waste. Sustainability.

[5] Kowalczewski P.Ł., Olejnik A., Rybicka I., Zielińska-Dawidziak M., Białas W., Lewandowicz G. (2021). Membrane filtration-assisted enzymatic hydrolysis affects the biological activity of potato juice. Molecules.

[6] Kieliszek M., Piwowarek K., Kot A.M., Pobiega K. (2020). The aspects of microbial biomass use in the utilization of selected waste from the agro-food industry. Open Life Sci..

[7] Kowalczewski P.Ł., Olejnik A., Białas W., Kubiak P., Siger A., Nowicki M., Lewandowicz G. (2019). Effect of Thermal Processing on Antioxidant Activity and Cytotoxicity of Waste Potato Juice. Open Life Sci..

[8] Kumar S., Kushwaha R., Verma M.L., Elsevier B.V. (2019). Recovery and Utilization of Bioactives from Food Processing Waste.

[9] Ancuţa P., Sonia A. (2020). Oil press-cakes and meals valorization through circular economy approaches: A review. Appl. Sci..

[10] Gupta A., Sharma R., Sharma S., Singh B., Prodyut Kumar P., Mahawar M.K., Abobatta W., Panja P. (2018). Oilseed as Potential Functional Food Ingredient. Trends & Prospects in Food Technology, Processing and Preservation.

[11] Adeleke B.S., Babalola O.O. (2020). Oilseed crop sunflower (*Helianthus annuus*) as a source of food: Nutritional and health benefits. Food Sci. Nutr..

[12] Chauhan V. (2021). Nutritional quality analysis of sunflower seed cake (SSC). Pharma Innov. J..

[13] Mirpoor S.F., Giosafatto C.V.L., Porta R. (2021). Biorefining of seed oil cakes as industrial co-streams for production of innovative bioplastics. A review. Trends Food Sci. Technol..

[14] Gültekin Subaşı B., Vahapoğlu B., Capanoglu E., Mohammadifar M.A. (2021). A review on protein extracts from sunflower cake: Techno-functional properties and promising modification methods. Crit. Rev. Food Sci. Nutr..

[15] Ahmar S., Gill R.A., Jung K.H., Faheem A., Qasim M.U., Mubeen M., Zhou W. (2020). Conventional and molecular techniques from simple breeding to speed breeding in crop plants: Recent advances and future outlook. Int. J. Mol. Sci..

[16] Jocić S., Miladinović D., Kaya Y. (2015). Breeding and Genetics of Sunflower.

[17] Pal D. (2011). Sunflower (Helianthus annuus L.) Seeds in Health and Nutrition.

[18] Romanić R. (2020). Cold Pressed Sunflower (*Helianthus annuus* L.) oil. Cold Pressed Oils.

[19] Islam R.T., Hossain M.M., Majumder K., Tipu A.H. (2016). In vitro Phytochemical Investigation of Helianthus annuus Seeds. Bangladesh Pharm. J..

[20] Anjum F.M., Nadeem M., Khan M.I., Hussain S. (2012). Nutritional and therapeutic potential of sunflower seeds: A review. Br. Food J..

[21] Ivanova P., Chalova V., Koleva L., Pishtiyski I. (2013). Amino acid composition and solubility of proteins isolated from sunflower meal produced in Bulgaria. Int. Food Res. J..

[22] Sarwar F. (2013). The role of oilseeds nutrition in human health: A critical review. J. Cereals Oilseeds.

[23] Savoire R., Lanoisellé J.L., Vorobiev E. (2013). Mechanical Continuous Oil Expression from Oilseeds: A Review. Food Bioprocess Technol..

[24] Ramadan M.F. (2020). Introduction to Cold Pressed Oils: Green Technology, Bioactive Compounds, Functionality, and Applications.

[25] Poiana M., Alexa E., Moigradean D., Popa M. The influence of the storage conditions on the oxidative stability and antioxidant properties of sunflower and pumpkin oil. Proceedings of the 44th Croatian & 4th International Symposium of Agriculture.

[26] Zoumpoulakis P., Sinanoglou V.J., Siapi E., Heropoulos G., Proestos C. (2017). Evaluating modern techniques for the extraction and characterisation of sunflower (*Hellianthus annus* L.) seeds phenolics. Antioxidants.

[27] Avni T.C.A., Anupriya S., Rai P., Maan K. (2016). Effects of Heating and Storage on Nutritional value of Sunflower Oil. DU J. Undergrad. Res. Innov..

[28] Nadeem M., Situ C., Mahmud A., Khalique A., Imran M., Rahman F., Khan S. (2014). Antioxidant activity of sesame (*Sesamum indicum* L.) cake extract for the stabilization of olein based butter. JAOCS J. Am. Oil Chem. Soc..

[29] Nandha R., Singh H., Garg K., Rani S. (2014). Therapeutic Potential of Sunflower Seeds: An Overview. Int. J. Res. Dev. Pharm. Life Sci..

[30] Bochkarev M.S., Egorova E.Y., Reznichenko I.Y., Poznyakovskiy V.M. (2016). Reasons for the ways of using oilcakes in food industry. Foods Raw Mater..

[31] Alagawany M., Farag M.R., El-Hack M.E.A., Dhama K. (2015). The Practical Application of Sunflower Meal in Poultry Nutrition. Adv. Anim. Vet. Sci..

[32] Serrapica F., Masucci F., Raffrenato E., Sannino M., Vastolo A., Barone C.M.A., Di Francia A. (2019). High fiber cakes from mediterranean multipurpose oilseeds as protein sources for ruminants. Animals.

[33] Nang Thu T.T., Bodin N., Saeger S., Larondelle Y., Rollin X. (2011). Substitution of fish meal by sesame oil cake (*Sesamum indicum* L.) in the diet of rainbow trout (*Oncorhynchus mykiss* W.). Aquac. Nutr..

[34] Wanjari N., Waghmare J. (2015). Phenolic and antioxidant potential of sunflower meal. Pelagia Res. Libr. Adv. Appl. Sci. Res..

[35] Lazaro E., Benjamin Y., Robert M. (2014). The Effects of Dehulling on Physicochemical Properties of Seed Oil and Cake Quality of Sunflower. Tanzania J. Agric. Sci..

[36] Araujo M.E.V., Barbosa E.G., Gomes F.A., Teixeira I.R., Lisboa C.F., Araújo R.S.L., Corrêa P.C. (2018). Physical properties of sesame seeds harvested at different maturation stages and thirds of the plant. Chil. J. Agric. Res..

[37] Ortiz-Hernandez A.A., Araiza-Esquivel M., Delgadillo-Ruiz L., Ortega-Sigala J.J., Durán-Muñoz H.A., Mendez-Garcia V.H., Yacaman M.J., Vega-Carrillo H.R. (2020). Physical characterization of sunflower seeds dehydrated by using electromagnetic induction and low-pressure system. Innov. Food Sci. Emerg. Technol..

[38] Igbozulike A.O., Amamgbo N. (2019). Effect of Moisture Content on Physical Properties of Fluted Pumpkin Seeds. J. Biosyst. Eng..

[39] Malik M.A., Saini C.S. (2016). Engineering properties of sunflower seed: Effect of dehulling and moisture content. Cogent Food Agric..

[40] Munder S., Argyropoulos D., Müller J. (2017). Class-based physical properties of air-classified sunflower seeds and kernels. Biosyst. Eng..

[41] De Figueiredo A.K., Baümler E., Riccobene I.C., Nolasco S.M. (2011). Moisture-dependent engineering properties of sunflower seeds with different structural characteristics. J. Food Eng..

[42] Costa C., Antonucci F., Pallottino F., Aguzzi J., Sun D.W., Menesatti P. (2011). Shape Analysis of Agricultural Products: A Review of Recent Research Advances and Potential Application to Computer Vision. Food Bioprocess Technol..

[43] Mirzabe A.H., Khazaei J., Chegini G.R. (2012). Physical properties and modeling for sunflower seeds. Agric. Eng. Int. CIGR J..

[44] Seiler G.J., Gulya T.J. (2016). Sunflower: Overview.

[45] Menzel C. (2020). Improvement of starch films for food packaging through a three-principle approach: Antioxidants, cross-linking and reinforcement. Carbohydr. Polym..

[46] Casoni A.I., Gutierrez V.S., Volpe M.A. (2019). Conversion of sunflower seed hulls, waste from edible oil production, into valuable products. J. Environ. Chem. Eng..

[47] Dobrzaski B., Stpniewski A. (2013). Physical Properties of Seeds in Technological Processes. Adv. Agrophys. Res..

[48] Rodríguez M., Nolasco S., Izquierdo N., Mascheroni R., Madrigal M.S., Flores D.C., Ramos A.Q. (2019). Microwave-assisted extraction of antioxidant compounds from sunflower hulls. Heat Mass Transf..

[49] Çetin N., Karaman K., Beyzi E., Sağlam C., Demirel B. (2021). Comparative Evaluation of Some Quality Characteristics of Sunflower Oilseeds (*Helianthus annuus* L.) Through Machine Learning Classifiers. Food Anal. Methods.

[50] Muttagi G.C., Joshi N. (2020). Physico-chemical composition of selected sunflower seed cultivars. Int. J. Chem. Stud..

[51] Akkaya M.R. (2018). Prediction of fatty acid composition of sunflower seeds by near-infrared reflectance spectroscopy. J. Food Sci. Technol..

[52] Nadeem M., Anjum F.M., Arshad M.U., Hussain S. (2010). Chemical characteristics and antioxidant activity of different sunflower hybrids and their utilization in bread. Afr. J. Food Sci..

[53] Kolláthová R., Varga B., Ivanišová E., Bíro D., Rolinec M., Juráček M., Šimko M., Gálik B. (2019). Mineral Profile Analysis of Oilseeds and Their By-Products As Feeding Sources for Animal Nutrition. Slovak J. Anim. Sci.

[54] Jafari S., Khazaei J., Arabhosseini A., Massah J., Khoshtaghaza M.H. (2011). Study on Mechanical Properties of Sunflower Seeds. Food Sci. Technol..

[55] Gupta R.K., Das S.K. (1997). Physical properties of sunflower seeds. J. Agric. Eng. Res..

[56] Sumon M.M., Tinggi S., Ekonomi I., Surabaya P., Hossain A. (2021). Comparative Study on Physicochemical Composition of Different Genotypes of Sunflower Seed and Mineral Profile of Oil Cake. Agriculturists.

[57] Pawar V.D., Patil J.N., Sakhale B.K., Agarkar B.S. (2001). Studies on selected functional properties of defatted sunflower meal and its high protein products. J. Food Sci. Technol..

[58] Taha F.S., Mohamed G.F., Mohamed S.H., Mohamed S.S., Kamil M.M. (2011). Optimization of the Extraction of Total Phenolic Compounds from Sunflower Meal and Evaluation of the Bioactivities of Chosen Extracts. Am. J. Food Technol..

[59] Rosa P.M., Antoniassi R., Freitas S.C., Bizzo H.R., Zanotto D.L., Oliveira M.F., Castiglioni V.B.R. (2009). Chemical composition of brazilian sunflower varieties. Helia.

[60] Žilic S., Barac M., Pešic M., Crevar M., Stanojevic S., Nišavic A., Saratlic G., Tolimir M. (2010). Characterization of sunflower seed and kernel proteins. Helia.

[61] Santalla E.M., Mascheroni R.H. (2003). Note: Physical Properties of High Oleic Sunflower Seeds. Food Sci. Technol. Int..

[62] Abdullah M.H.R.O., Ch’ng P.E., Lim T.H. (2011). Some Physical Properties of Parkia Speciosa Seeds. Int. Conf. Food Eng. Biotechnol..

[63] Krajewska M., Ślaska-Grzywna B., Andrejko D. (2016). Physical Properties of Seeds of the Selected Oil Plants. Agric. Eng..

[64] Coşkuner Y., Gökbudak A. (2016). Dimensional specific physical properties of fan palm fruits, seeds and seed coats (*Washingtonia robusta*). Int. Agrophys..

[65] Aviara N.A., Gwandzang M.I., Haque M.A. (1999). Physical properties of guna seeds. J. Agric. Eng. Res..

[66] Niveditha V.R., Sridhar K.R., Balasubramanian D. (2013). Physical and mechanical properties of seeds and kernels of canavalia of coastal sand dunes. Int. Food Res. J..

[67] Seifi M.R., Alimardani R. (2010). Moisture-Dependent Physical Properties of Sunflower (SHF8190). Mod. Appl. Sci..

[68] Babić L.J., Radojčin M., Pavkov I., Babić M. (2012). The physical and compressive load properties of sunflower (*Helianthus annuus* L.) fruit. Helia.

[69] Popović S., Hromiš N., Šuput D., Bulut S., Romanić R., Lazić V. (2020). Valorization of By-Products From the Production of Pressed Edible Oils to Produce Biopolymer Films. Cold Pressed Oils.

[70] Sobczak P., Zawislak K., Starek A., Zukiewicz-Sobczak W., Sagan A., Zdybel B., Andrejko D. (2020). Compaction process as a concept of press-cake production from organic waste. Sustainability.

[71] Adesina S.A. (2019). Effect of processing on the proximate composition of sunflower (*Helianthus annuus*) seeds. Agro-Science.

[72] Rani R., Badwaik L.S. (2021). Functional Properties of Oilseed Cakes and Defatted Meals of Mustard, Soybean and Flaxseed. Waste Biomass Valorization.

[73] Sinkovič L., Kolmanič A. (2021). Elemental composition and nutritional characteristics of cucurbita pepo subsp. Pepo seeds, oil cake and pumpkin oil. J. Elem..

[74] Sunil L., Appaiah P., Prasanth Kumar P.K., Gopala Krishna A.G. (2015). Preparation of food supplements from oilseed cakes. J. Food Sci. Technol..

[75] Cozea A., Ionescu N., Popescu M., Neagu M., Gruia R. (2016). Comparative study concerning the composition of certain oil cakes with phytotherapeutical potential. Rev. Chim..

[76] Hussain S., Jõudu I., Bhat R. (2020). Dietary fiber from underutilized plant resources-A positive approach for valorization of fruit and vegetable wastes. Sustainability.

[77] Dhingra D., Michael M., Rajput H., Patil R.T. (2012). Dietary fibre in foods: A review. J. Food Sci. Technol..

[78] Maphosa Y., Jideani V.A. (2016). Dietary fiber extraction for human nutrition—A review. Food Rev. Int..

[79] Nevara G.A., Kharidah S., Muhammad S., Zawawi N., Mustapha N.A., Karim R. (2021). Dietary Fiber: Fractionation, Characterization and Potential Sources from Defatted Oilseeds. Foods.

[80] Bhise S.R., Kaur A., Manikantan M.R., Singh B. (2015). Development of textured defatted sunflower meal by extrusion using response surface methodology. Acta Aliment..

[81] Bhise S., Kaur A. (2015). The effect of extrusion conditions on the functional properties of defatted cake of sunflower-maize based expanded snacks. Int. J. Food Ferment. Technol..

[82] Brachet M., Arroyo J., Bannelier C., Cazals A., Fortun-Lamothe L. (2015). Hydration capacity: A new criterion for feed formulation. Anim. Feed Sci. Technol..

[83] Lin M.J., Humbert E.S., Sosulski F.W. (1974). Functional Properties Sunflower Meal Products of. J. Food Sci..

[84] Sosulski F., Fleming S.E. (1977). Chemical, functional, and nutritional properties of sunflower protein products. J. Am. Oil Chem. Soc..

[85] Grasso S., Omoarukhe E., Wen X., Papoutsis K., Methven L. (2019). The use of upcycled defatted sunflower seed flour as a functional ingredient in biscuits. Foods.

[86] Amza T., Amadou I., Zhu K.X., Zhou H.M. (2011). Effect of extraction and isolation on physicochemical and functional properties of an underutilized seed protein: Gingerbread plum (Neocarya macrophylla). Food Res. Int..

[87] White N.D.G., Jayas D.S. (2001). Physical properties of canola and sunflower meal pellets. Can. Biosyst. Eng..

[88] Dabbour M., He R., Ma H., Musa A. (2018). Optimization of ultrasound assisted extraction of protein from sunflower meal and its physicochemical and functional properties. J. Food Process Eng..

[89] Cai T., Chang K.C., Lunde H. (1996). Physicochemical Properties and Yields of Sunflower Protein Enzymatic Hydrolysates As Affected by Enzyme and Defatted Sunflower Meal. J. Agric. Food Chem..

[90] Melo D., Álvarez-Ortí M., Nunes M.A., Costa A.S.G., Machado S., Alves R.C., Pardo J.E., Oliveira M.B.P.P. (2021). Whole or defatted sesame seeds (*Sesamum indicum* L.)? The effect of cold pressing on oil and cake quality. Foods.

[91] Arrutia F., Binner E., Williams P., Waldron K.W. (2020). Oilseeds beyond oil: Press cakes and meals supplying global protein requirements. Trends Food Sci. Technol..

[92] Jannathulla R., Dayal J.S., Ambasankar K., Muralidhar M. (2018). Effect of Aspergillus niger fermented soybean meal and sunflower oil cake on growth, carcass composition and haemolymph indices in Penaeus vannamei Boone, 1931. Aquaculture.

[93] Zentek J., Knorr F., Mader A. (2013). Reducing Waste in Fresh Produce Processing and Households through Use of Waste as Animal Feed.

[94] Soetan K.O., Olaiya C.O., Oyewole O.E. (2010). The importance of mineral elements for humans, domestic animals and plants: A review. Afr. J. Food Sci..

[95] Njuguna D.G., Wanyoko J.K., Kinyanjui T., Wachira F.N. (2013). Mineral Elements in the Kenyan Tea Seed Oil Cake. Int. J. Res. Chem. Environ..

[96] Chaves E.S., dos Santos E.J., Araujo R.G.O., Oliveira J.V., Frescura V.L.A., Curtius A.J. (2010). Metals and phosphorus determination in vegetable seeds used in the production of biodiesel by ICP OES and ICP-MS. Microchem. J..

[97] Goiri I., Zubiria I., Benhissi H., Atxaerandio R., Ruiz R., Mandaluniz N., Garcia-Rodriguez A. (2019). Use of cold-pressed sunflower cake in the concentrate as a low-input local strategy to modify the milk fatty acid profile of dairy cows. Animals.

[98] Zubiria I., Garcia-Rodriguez A., Atxaerandio R., Ruiz R., Benhissi H., Mandaluniz N., Lavín J.L., Abecia L., Goiri I. (2019). Effect of feeding cold-pressed sunflower cake on ruminal fermentation, lipid metabolism and bacterial community in dairy cows. Animals.

[99] Hansen J.Ø., Skrede A., Mydland L.T., Øverland M. (2017). Fractionation of rapeseed meal by milling, sieving and air classification—Effect on crude protein, amino acids and fiber content and digestibility. Anim. Feed Sci. Technol..

[100] Chetrariu A., Dabija A. (2021). Quality Characteristics of Spelt Pasta Enriched with Spent Grain. Agronomy.

[101] Omowaye-Taiwo O.A., Fagbemi T.N., Ogunbusola E.M., Badejo A.A. (2015). Effect of germination and fermentation on the proximate composition and functional properties of full-fat and defatted cucumeropsis mannii seed flours. J. Food Sci. Technol..

[102] Onipe O.O., Beswa D., Jideani A.I.O. (2017). Effect of size reduction on colour, hydration and rheological properties of wheat bran. Food Sci. Technol..

[103] Coțovanu I., Batariuc A., Mironeasa S. (2020). Characterization of quinoa seeds milling fractions and their effect on the rheological properties of wheat flour dough. Appl. Sci..

[104] Iyenagbe D.O., Malomo S.A., Idowu A.O., Badejo A.A., Fagbemi T.N. (2017). Effects of thermal processing on the nutritional and functional properties of defatted conophor nut (Tetracarpidium conophorum) flour and protein isolates. Food Sci. Nutr..

[105] Konak M., Çarman K., Aydin C. (2002). Physical properties of chick pea seeds. Biosyst. Eng..

[106] Dabadé D.S., Jacxsens L., Miclotte L., Abatih E., Devlieghere F., De Meulenaer B. (2021). Survey of multiple biogenic amines and correlation to microbiological quality and free amino acids in foods. Food Control.

[107] Gifty A.G., De Meulenaer B., Olango T.M. (2018). Variation in tuber proximate composition, sugars, fatty acids and amino acids of eight Oromo dinich (Plectranthus edulis) landraces experimentally grown in Ethiopia. J. Food Compos. Anal..

[108] Dulf F.V. (2012). Fatty acids in berry lipids of six sea buckthorn (*Hippophae rhamnoides* L., subspecies carpatica) cultivars grown in Romania. Chem. Cent. J..

